# A novel paclitaxel coated balloon with increased drug transfer for treatment of complex vascular lesions

**DOI:** 10.1371/journal.pone.0259106

**Published:** 2021-10-29

**Authors:** Ole Gemeinhardt, Beatrix Schnorr, Ulrich Speck, Bruno Scheller

**Affiliations:** 1 Department of Radiology, Charité –Universitätsmedizin Berlin, Corporate Member of Freie Universität Berlin and Humboldt-Universität zu Berlin, Berlin, Germany; 2 Clinical and Experimental Interventional Cardiology, University of Saarland, Homburg/Saar, Germany; Medical University Innsbruck, AUSTRIA

## Abstract

**Background:**

Drug coated balloons (DCB) with paclitaxel (Ptx) dose of 2–3.5 μg/mm^2^ balloon surface inhibit restenosis with different effectiveness and duration of success. A clinical dose finding study is not known for any of the currently marketed products. The aim of the present preclinical trial was to investigate a novel DCB coated with 6 μg Ptx/mm^2^ in a porcine model.

**Methods and results:**

The current study investigated a DCB with a novel, modified iopromide based matrix with 6 μg Ptx/mm^2^. Drug transfer to the vessel wall of peripheral arteries was compared with a dose of 3 μg Ptx/mm^2^ and two fully overlapping DCB with 3 μg Ptx/mm^2^, each.

Ptx concentration in the vessel wall after drug transfer was about twice as high for balloons with 6 μg/mm^2^ (1957±1472 μg/g) and two overlapping DCB with 3 μg Ptx/mm^2^, each (1287±619 μg/g) compared to a single balloon with 3 μg Ptx/mm^2^, (787±738 μg/g), with statistical significant differences for 1x6 μg/mm^2^ vs. 1x3 μg/mm^2^ (p = 0.017) but not for 2x3 μg/mm^2^ vs. 1x3 μg/mm^2^ (p = 0.184) and 1x6 μg/mm^2^ vs. 2x3 μg/mm^2^ (p = 0.178). The proportion of residual Ptx on balloon after treatment was similar for all groups between 6±1% and 10±3% of dose on balloon.

**Conclusion:**

The dose of 6 μg Ptx/mm^2^ was successfully as well as reproducibly coated on conventional balloon catheters. Increased Ptx on balloons resulted in increased drug concentration in the vessel wall. A single balloon with 6 μg Ptx/mm^2^ seems to provide double dose compared to 3 μg Ptx/mm^2^, facilitates the procedure, and may reduce medico-economic cost compared to the use of two standard DCB.

## Introduction

Vascular diseases such as atherosclerosis and its secondary diseases are the cause of many serious conditions in middle and old age and the leading cause of death in western countries [1]. More than 200 million people worldwide suffer from peripheral artery disease (PAD) alone, with increasing tendency [2]. Endovascular treatment of vascular diseases (e.g. balloon angioplasty, atherectomy and/or stent implantation) has become established in many indications and offers immediately effective and less invasive alternatives [3]. This applies to the treatment of arterial occlusive disease in coronary and peripheral arteries. In this case, drug-coated medical devices (stents and/or balloon catheters) have led to an improvement in the durability of interventions, thus reducing the need to repeat treatments leading to substantial cost savings or to carry out more invasive measures [3].

In peripheral arteries clinical studies have shown improved primary patency and reduction of repeated interventions by drug coated balloons (DCB) compared to conventional balloon angioplasty [4–8]. DCB used in these former trials include rather lower dosages for paclitaxel between only 2 μg/mm^2^ balloon surface up to the highest dosed product with 3.5 μg/mm^2^. Meta-analyses of various DCB studies suggest that trials using DCB with a nominal paclitaxel dose of 2 μg/mm^2^ were associated with a less marked treatment effect favoring DCB with a paclitaxel dose of > 3 μg/mm^2^ [9,10] although contradictory results have been published for different products [11]. Nevertheless, even after treatment with DCB with 3.5 μg/mm^2^ there are therapy failures that do not respond to treatment or where the treatment effect such as prevention of restenosis of the treated vessel does not last long.

In the recently published COPA CABANA study Tepe et al. [12] found in a non-randomized fashion of patients and lesions, that after application of 2 fully overlapping DCB with 3 μg Ptx/mm^2^ balloon surface each in repeated restenotic in-stent restenosis of superficial femoral and popliteal arteries, the late lumen loss was smaller after 6 months, than after treatment with a single dose or POBA (double dose 0.11 ± 0.78 mm, single dose 0.34 ± 1.12 mm, POBA 1.58 ± 1.10 mm). After 24 months, there were no TLR in the 22 patients of the double-dose group, whereas the TLR even in the single-dose DCB group were 52%. In some patients with very long lesions, longitudinal overlap of DCBs was required, resulting in deployment of up to 4 DCBs within 1 short vessel segment during a single intervention. None of the patients who underwent follow-up angiography had side effects such as aneurysm. The double dose for treating recurrent ISR did not cause recognizable adverse events up to 24 months [12]. These results suggest that treatment with a paclitaxel dose of ≥ 6 μg/mm^2^ might improve therapy in difficult to treat lesions.

Commonly used drug dosages on DCB were based on the balloon surface, since long balloons with a large diameter require more active ingredient to treat long segments of large vessels than short segments of small-lumen vessels. The originally introduced paclitaxel dose of 3 μg/mm^2^ [13,14] was the maximum feasible loading of smooth balloon membranes at that time. Now, we developed a paclitaxel formulation that enabled coating a DCB with 6 μg/mm^2^ balloon surface.

The aim of the current study was to investigate a novel balloon catheter with a paclitaxel dose of 6 μg/mm^2^ and provide information whether coating one balloon with 6 μg Ptx/mm^2^ affects the drug transfer to the vessel wall of peripheral arteries compared to 3 μg Ptx/mm^2^ (same formulation and coating method). PTA balloon catheters were coated with an adjusted Paccocath formulation [13,15]. Application of one balloon coated with 6 μg Ptx/mm^2^ balloon surface (1 x double dose) was compared to application of two fully overlapping balloons, each coated with 3 μg Ptx/mm^2^ (2 x single dose), and application of one balloon coated with 3 μg Ptx/mm^2^ (1 x single dose).

## Methods

### Balloon catheters and drug content

Conventional angioplasty balloon catheters with four different sizes (4.0 x 40 mm, 5.0 x 40 mm, 6.0 x 40 mm, 7.0 x 40 mm) were coated with 3 μg Ptx/mm^2^ or 6 μg Ptx/mm^2^ using a novel modified Paccocath coating including iopromide as excipient. Coated balloon catheters were sterilized.

### Paclitaxel (Ptx) content on balloons

The balloons were inflated in cryovials for analyzing paclitaxel. Inflated balloons were extracted and paclitaxel content was determined by HPLC/UV analysis as described below.

### Drug transfer to the vessel wall

The in vivo study was performed in 6 castrated male domestic pigs (body weight 27.2 ± 1.2 kg, about three months old). All animal studies were conducted at the Institute of Medical Technology and Research (IMTR GmbH, Rottmersleben, Germany) in accordance with the guidelines of the European commission directive 86/609/EEC and the German Animal Protection Act based upon the Animal Ethics Committee approvals (Saxony–Anhalt, Germany).

### Anesthesia and pre-interventional procedure

Two days before the treatment dual platelet therapy was administered including 75 mg Clopidogrel and 100 mg Acetylsalicylic acid. Long-acting Verapamil hydrochloride was given within 24 hours prior to the procedure to prevent vascular spasm during the procedure.

The pigs were sedated with 0.2 ml/kg ketamine (Ursotamin®, Serumwerk Bernburg, Germany) plus 0.1 ml/kg xylazinhydrochloride 2% (Xylazin®, Riemser Arzneimittel GmbH, Germany) before general anesthesia was induced with intravenous administration of propofol (3 mg/kg, Recofol 1%, Curamed Pharma GmbH, Germany) followed by intramuscular administration of 0.4 mg/kg Meloxicam (Metacam®, Boehringer Ingelheim Vet Medica, Ingelheim Rhein, Germany) and intravenous administration of 0,1 mg/kg Butorphanol (Morphasol, aniMedica GmbH, Germany) as analgetic. The pigs were intubated (Endonorm, Rüsch GmbH, Germany) and ventilation was started using a mixture of 30–60 vol% of pure oxygen, 40–70 vol% air and 1–2 vol% of isoflurane (Isofluran Curamed, Curamed Pharma GmbH, Germany).

Meloxicam (0.4 ml/kg) and Butorphanol (0.1 mg/kg) were administered as analgetic. A common carotid artery was surgically exposed and an intra-arterial sheath (Avanti +, 8F, Cordis, USA) was introduced. Heparin-Natrium 5000 IU and 250 mg DL-lysine mono(acetylsalicylate) were administered intra-arterially as a bolus. Under fluoroscopic control a guiding catheter (Launcher JL 3.5, 6F, Medtronic, USA) was introduced through the arterial sheath over a guide wire. Angiography of internal and external iliac arteries was performed before and after treatment using a Siemens AXIOM Artis zee fluoroscope. External and internal iliac arteries were visualized using iopromide (Ultravist-370, BSP AG, Germany) as contrast agent. Suitable vessel segments for DCB deployment were selected in the assigned arteries. Throughout the procedure blood pressure, electrocardiogram, oxygenation, and temperature were monitored continuously.

### Interventional procedure in internal and external iliac arteries

After selection of a suitable vessel segment of the internal iliac artery a marker stent (Coroflex Blue NEO, 4.0 x 13 mm, B.Braun Melsungen AG, Germany) was implanted distal of the selected segment to assure proper vessel dissection at the end of the study. The coated balloons were introduced and deployed. Internal iliac arteries were treated with balloons sized 4.0 x 40 mm or 5.0 x 40 mm and external iliac arteries with balloons sized 6.0 x 40 mm or 7.0 x 40 mm, respectively. Each vessel segment was treated with one (1x3 μg/mm^2^ or 1x6 μg/mm^2^) or two (2x3 μg/mm^2^) coated balloons, applying appropriate inflation pressure to achieve overstretch of about 20% versus the reference diameter of the vessel. The duration of inflation was 60 seconds. Afterwards, the balloons were deflated and retracted. For treatment of 2x3 μg/mm^2^, a second DCB was placed in the same position as the first DCB, inflated for 60 seconds, deflated and retracted. All balloons were collected for residual drug extraction and quantification.

Afterwards the animals were euthanized in deep anesthesia using supersaturated potassium chloride. The treated vessel segments including the distal marker stents (internal iliac artery) were dissected for drug analysis. For extraction a defined volume of ethanol was added to achieve an ethanol concentration of ≥ 80%. The samples were homogenized (Precelly 24 Dual Homogenizer, PEQLAB Biotechnologie GmbH, Erlangen, Germany) and extracted by 30 min treatment with ultrasound at room temperature and then centrifuged for 10 minutes at 17,500 g.

### Quantification of paclitaxel

Paclitaxel was quantified by HPLC with UV detection (Shimadzu Nexera-i lc-2040c 3D, Shimadzu Corporation, Kyoto, Japan). Column: C18, 5 μm, 25 cm x 4.6 mm. Mobile phase: 45% phosphate buffer 0.005 M and 55% acetonitrile, 1 ml/min. Detection: 230 nm. Column temperature: 35°C. At least 20 μl of the supernatant was injected into the HPLC unit. A standard curve (paclitaxel) was established during the same run (concentration between 5 and 500 μg/ml).

### Statistical analysis

Data are presented as mean ± SD. Differences between suitable pairs of data are assessed by Student’s t-test (2-tailed), except differences for drug transfer to tissue. Statistical significance was assumed at p ≤ 0.05. Analysis of differences in drug transfer to tissue was performed using a mixed model with repeated measures (MMRM). The total amount of Ptx transfer to tissue was used as a dependent variable in a first model, and the relative Ptx transfer as % of dose on balloon in a second model. The dosing scheme and the balloon size were fixed factors. The animal constituted a repeated factor in the model, a compound symmetry covariance structure was utilized. A type 3 test of fixed effects was used to assess the statistical significance of the fixed factors ‘dose’ and ‘balloon size’. Differences between factor levels were evaluated based on estimated least square means. Computations of differences in drug transfer were performed using the SAS version 9.4 software system (SAS Institute Inc., Cary, NC, USA).

## Results

### Paclitaxel (Ptx) content on balloons and technical performance

**Fig 1:**
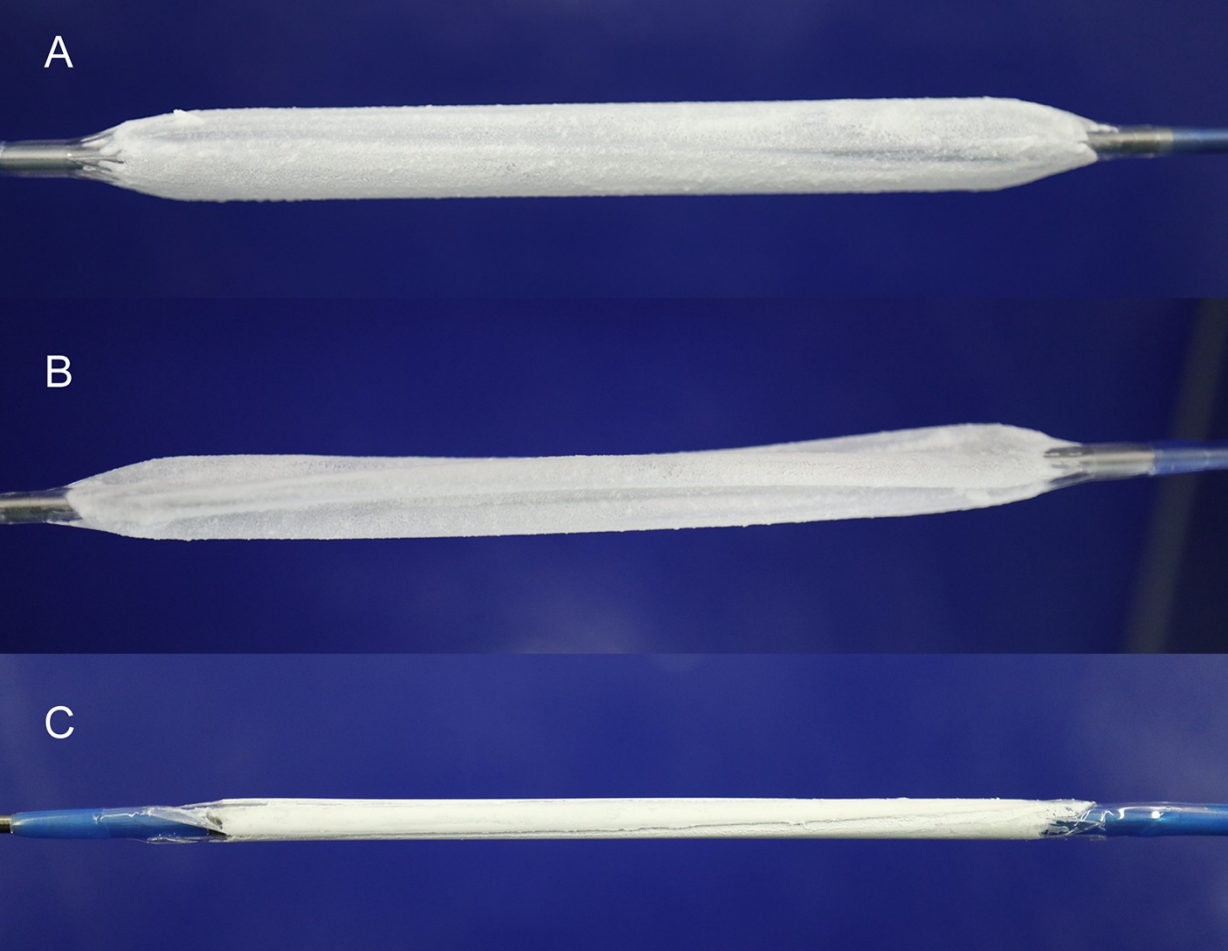
Conventional angioplasty balloon catheters coated with a novel, modified iopromide based matrix. Drug dose: 6 μg Paclitaxel/mm^2^ balloon surface. Balloon size: 5.0 x 40 mm. (A) inflated, (B) partly inflated with three folds, and (C) folded.

Mean Ptx doses on balloons coated with a single dose or a double dose were 3.1 ± 0.1 μg/mm^2^ and 5.9 ± 0.2 μg/mm^2^ balloon surface, respectively (Table 1). Visual inspection showed homogenous coatings for all balloons (Fig 1). All balloons performed technically as intended.

**Table 1:** Ptx doses on balloons coated with single dose or double dose.

**3 μg Ptx/mm**^**2**^ **balloon surface (nominal)**				
Balloon size	4x40	5x40	6x40	7x40
Ptx dose on balloon [μg/mm^2^]	3.2	3.0	3.2	3.0
	3.0	3.1	3.1	2.9
	3.2	3.2	3.1	2.9
	3.0	2.9	3.0	3.1
Mean ± SD	3.1 ± 0.1	3.1 ± 0.1	3.1 ± 0.1	3.0 ± 0.1
Mean ± SD (all sizes)	3.1 ± 0.1			
**6 μg Ptx/mm**^**2**^ **balloon surface (nominal)**				
Balloon size	4x40	5x40	6x40	7x40
Ptx dose on balloon [μg/mm^2^]	5.8	6.1	5.6	5.7
	6.0	5.9	6.1	5.7
	5.7	6.1	6.1	5.7
Mean ± SD	5.8 ± 0.2	6.0 ± 0.1	5.9 ± 0.3	5.7 ± 0.0
Mean ± SD (all sizes)	5.9 ± 0.2			

### In-vivo drug transfer to the vessel wall

No device failures or device-related animal morbidity or mortality occurred during the study. Neither target-site thrombi or thrombemboli nor device related abnormalities in electrocardiogram were observed in any of the treatment groups. Blood pressure (taken before and directly after intervention) remained stable during procedure (- 1.5 ± 19.5 mmHg). Two treated internal iliac arteries showed perivascular hemorrhages in post mortem dissection. One artery was treated with a single dose and the other one with 2 x single dose. No other device-related findings were discovered at necropsy. Balloons were inflated to an estimated oversize ratio of approximately 1.2. Inflation pressure was 8–14 atm (1x3 μg/mm^2^ 10.5 ± 1.8 atm, 2x3 μg/mm^2^ 11.3 ± 1.6 atm, 1x6 μg/mm^2^ 10.8 ± 1.8 atm) (Table 2).

**Table 2:** Inflation pressure, Paclitaxel transferred to vessel wall and residual paclitaxel on balloon after treatment.

Group	Balloon size	Inflation pressure*	Drug transfer to tissue			Residual Ptx on used balloon, % of dose on balloon*
			Ptx	Ptx concentration in tissue	Ptx in tissue, % of dose on balloon*	
	[mm]	[Atm]	[μg]	[ng/mg = μg/g]	[%]	[%]
1x3	4.0x40	8	216	498	12.0	13.9
	4.0x40	14	262	792	14.6	9.2
	5.0x40	10	621	1257	27.6	8.7
	5.0x40	10	652	333	29.0	8.4
	6.0x40	12	71	182	2.5	6.4
	6.0x40	10	118	270	4.2	10.1
	7.0x40	10	327	559	10.2	11.7
	7.0x40	10	769	2405	24.0	7.7
**Mean ± SD**		**10.5 ± 1.8**	**379 ± 265**	**787 ± 738**	**15.5 ± 10.3**	**9.5 ± 2.4**
2x3	4.0x40	14 / 14	374	661	10.4	18.8 / 8.9
	4.0x40	12 / 12	564	1078	15.7	7.3 / 8.5
	5.0x40	12 / 12	963	2131	21.4	10.0 / 7.8
	5.0x40	10 / 10	1682	1898	37.4	8.3 / 9.3
	6.0x40	10 / 10	298	978	5.3	13.7 / 8.4
	6.0x40	10 / 10	418	1265	7.4	8.8 / 8.3
	7.0x40	12 / 14	249	427	3.9	6.5 / 7.9
	7.0x40	10 / 10	1001	1861	15.6	11.9 / 12.7
**Mean ± SD**		**11.3 ± 1.6**	**694 ± 492**	**1287 ± 619**	**14.6 ± 10.9**	**9.8 ± 3.1**
1x6	4.0x40	8	545	1653	16.2	6.5
	4.0x40	12	395	721	11.7	5.8
	5.0x40	10	1476	4515	33.1	4.1
	5.0x40	10	1424	1864	32.0	6.4
	6.0x40	12	242	757	4.5	4.3
	6.0x40	10	414	1275	7.7	5.4
	7.0x40	14	363	911	5.9	7.1
	7.0x40	10	1509	3957	24.5	4.1
**Mean ± SD**		**10.8 ± 1.8**	**796 ± 564**	**1957 ± 1472**	**16.9 ± 11.5**	**5.5 ± 1.2**

* In the 2x3 group, values for inflation pressure and residual paclitaxel on used balloons are given for both balloons used in one arterial segment.

Paclitaxel per g tissue was about twice (2x3 μg/mm^2^) or 2.5 times (1x6 μg/mm^2^) for double dose application compared to single dose (Table 3). The dose 1x6 μg/mm^2^ attains significantly higher concentrations as compared to the standard dose, whereas the dose 2x3 μg/mm^2^ missed significance compared to the standard dose and to the 1x6 μg/mm^2^ dose. The absolute transfer of paclitaxel to the vessel wall confirms the results, showing statistical significance for both double dose groups compared to the single dose. No significant difference was found between the two double doses. Drug transfer to vessel wall in % of dose on balloon was similar for double dose and single dose application.

**Table 3:** Paclitaxel transferred to vessel wall and residual paclitaxel on balloon after treatment–summarized for doses.

	Dose [μg/mm^2^]			p-value
	1x3	2x3	1x6	
n treated arteries	8	8	8	
**Drug transfer to tissue**				
Ptx Mean ± SD [μg]	379 ± 265	694 ± 492	796 ± 564	1x3 vs 1x6 = 0.002 2x3 vs 1x3 = 0.003 2x3 vs 1x6: ns
Ptx concentration in tissue Mean ± SD [ng/mg = μg/g]	787 ± 738	1287 ± 619	1957 ± 1472	1x3 vs 1x6 = 0.02 2x3 vs 1x3: ns 2x3 vs 1x6: ns
Ptx in tissue, % of dose on balloon* Mean ± SD [%]	15.5 ± 10.3	14.6 ± 10.9	16.9 ± 11.5	ns
**Residual Ptx on used balloons**				
% of dose on balloon** Mean ± SD [%]	9.5 ± 2.4	9.8 ± 3.1	5.5 ± 1.2	1x3 vs 1x6 = <0.001 2x3 vs 1x3: ns 2x3 vs 1x6 = <0.001

* Values of balloons with 2x3 μg/mm^2^ are given with regard to dose on both balloons.

** n = 16; Values of balloons with 2x3 μg/mm^2^ are given with regard to dose on each balloon with 3 μg/mm^2^. ns: not significant.

Drug transfer to internal iliac artery was significantly higher compared to external iliac artery (p = 0.002) (Table 4). Larger diameter balloons showed higher drug transfer in % of dose on balloon to vessel wall compared to balloons with smaller diameters in each vessel beyond all three dose groups (1x3, 1x6 and 2x3 μg/mm^2^) (Table 4). Residual Ptx amount per mm^2^ balloon surface after treatment was similar for all groups (0.3 ± 0.1 μg/mm^2^, n = 24) corresponding to values of dose on balloon between 5.5% and 9.5% (Table 3).

**Table 4:** Paclitaxel transferred to the vessel wall in % of dose on balloon–summarized for drug doses, balloon sizes and arteries.

Arteries	Balloon size (treated arteries per dose) mm x mm (n)	Drug transfer to tissue [% of dose on balloon]			
		1x3 μg/mm^2^	2x3 μg/mm^2^	1x6 μg/mm^2^	p-value
A. iliaca int.	4x40 (2)	13.3 ± 1.8	13.1 ± 3.7	13.9 ± 3.1	0.002
	5x40 (2)	28.3 ± 1.0	29.4 ± 11.3	32.5 ± 0.8	
A. iliaca ext.	6x40 (2)	3.4 ± 1.2	6.4 ± 1.5	6.1 ± 2.3	0.010
	7x40 (2)	17.1 ± 9.7	9.8 ± 8.3	15.2 ± 13.1	
A. iliaca int.	4.0 and 5.0 x 40 (4)	20.8 ± 8.7	21.2 ± 11.7	23.2 ± 10.9	0.002
A. iliaca ext.	6.0 and 7.0 x 40 (4)	10.2 ± 9.8	8.1 ± 5.2	10.7 ± 9.3	

P-values: 4x40 vs 5x40 and 6x40 vs 7x40 beyond all doses (n = 6 vs n = 6); A. iliaca int. vs A. iliaca ext. beyond all doses (n = 12 vs n = 12).

## Discussion

Effective drug transfer to the vessel wall is considered to be a precondition of inhibition of neointimal formation following interventional treatment with drug-eluting stents or DCB. Inadequate drug transfer leads to insufficient neointima inhibition. Various methods for increased drug transfer into the vessel wall and possibly improved efficacy have been discussed such as vessel preparation by scoring or cutting balloon catheters or the use of directional atherectomy [16]. Prolonged contact time of the DCB with the vessel wall potentially increases the drug transfer to the vessel wall but is not always applicable and also time consuming.

A potential approach to increase the amount of drug transfer to vessel wall and duration of drug efficacy is the application of a higher drug dose. A clinical dose finding study is not known for any of the currently marketed products. Data from the COPA CABANA trial indicate that a paclitaxel density on balloons higher than 3 μg/mm^2^ may be more efficacious in preventing repeated restenosis in difficult to treat lesions. Twenty two patients underwent two DCB treatments of the same vessel segment during a single procedure for re-recurrent restenosis following index treatment resulting in prolonged vessel expansion (ca. 5 min for 2 x DCB versus ca. 2 min for 1 x DCB or uncoated balloon) resulting in slightly reduced residual stenosis after treatment and a tendency towards smaller late lumen loss at 6 months compared to treatment with a single dose or POBA (double dose 0.11 ± 0.78 mm, single dose 0.34 ± 1.12 mm, POBA 1.58 ± 1.10 mm). The advantage was maintained until 24 month-follow-up: no TLR in the double-dose group vs 52% in the single-dose group [12]. To what extend two times balloon inflation and/or prolonged contact time to the arterial wall influenced the efficacy of therapy was not examined and cannot be derived from the study. Assuming that the increased dose might have an effect and with regard to medico-economic issues a DCB with a drug load of 6 μg Ptx/mm^2^ instead of using two DCB with 3 μg Ptx/mm^2^ might be useful. Currently, commercially available DCB are only available with a maximum paclitaxel dose of 3.5 μg/mm^2^ balloon surface.

The current study investigated a DCB with a novel, modified iopromide based matrix [14] with 6 μg Ptx/mm^2^. We compared the amount of drug transfer to the vessel wall of peripheral arteries with a single dose (3 μg Ptx/mm^2^) and a double dose of two DCB with 3 μg Ptx/mm^2^, each. For both balloon loading doses (3 μg and 6 μg Ptx/mm^2^) the same balloon platform, coating formulation and coating technology was used.

In the in vivo study no device related animal deaths, no in-life thrombi, thrombotic occlusions, or outflow obstructions were observed. There were no device-related alterations in ECG and blood pressure. Paclitaxel transfer to vessel wall in % of drug dose on balloon was about equal for double and single dose (15–17% of dose on balloon) resulting in about double amount of transferred paclitaxel to the vessel wall. The one minute inflation of the 1x6 μg/mm^2^ balloon reached a mean paclitaxel concentration in the iliac artery wall of almost 2000 ng/mg tissue, which is much higher compared to results presented in other studies [17–21].

Cremers et al. [18] investigated in peripheral arteries of pigs the drug transfer from balloon catheters with a nitinol constraining structure over the balloon (“Chocolate”) and a commercial DCB (IN.PACT Pacific, Medtronic, Dublin, Ireland), each coated with 3 μg Ptx/mm^2^. Drug transfer into the arterial wall after 2 min inflation time resulted in 381 ± 236 ng/mg tissue for the Chocolate and 276± 187 ng/mg for the IN.PACT Pacific. Lower numbers of drug transfer have been reported for a Shellac coating with a Ptx concentration of 143 ± 60 ng/mg after 1 min inflation time [19] as well as for a Ptx-Resveratrol coated DCB with 145 ± 50 ng/mg after 1 min inflation time [20]. Even lower drug transfer was shown for the Lutonix^®^ DCB coated with 2 μg Ptx/mm^2^ (59 ± 54 ng/mg) [17] and for a DCB based on the Passeo™ platform with drug loads of 1 μg Ptx/mm^2^ and 3 μg Ptx/mm^2^ (15 ± 21 ng/mg and 15 ± 16 ng/mg) [17], the latter two studies with just 30 sec inflation time.

Residual drug on balloon after treatment typically ranged from 8% to 23% of the balloon dose [17,18,20] and was highest with Shellac coating with 31% and 53% after 2 and 1 min balloon inflation [19]. In our study the residual amount of paclitaxel per mm^2^ balloon surface was between 5% and 10% of the dose on balloon. The mean drug concentration in arterial tissue was about double in internal iliac arteries compared to external iliac arteries. Furthermore, balloons with larger diameter showed higher drug transfer to vessel wall compared to balloons with smaller diameter in the same type of artery. This might indicate that a larger balloon-to-vessel diameter ratio could positively influence the drug transfer.

Despite the progress in endovascular treatment of patients with peripheral arterial disease, restenosis remains the major hinderance, particularly in patients with femoropopliteal lesions. Drug transfer to and long residence time of the drug in the arterial wall might play a substantial role for inhibition of neointimal proliferation and restenosis [22]. In the peripheral porcine model longer-term tissue residence time and higher levels of neointima inhibition have been demonstrated for higher dose (3.5 μg Ptx/mm^2^) compared to lower dose (2 μg Ptx/mm^2^) DCB [23].

Another complex indication is the treatment of stenosed or occluded hemodialysis arteriovenous fistulae (AV shunts). In the Lutonix AV Randomized Trial, treatment with the 2 μg Ptx/mm^2^ coating led to fewer re-interventions than POBA to maintain target lesion patency up to 9 months, but not at later time points [24]. In the In.Pact AV Access study primary patency was significantly higher after treatment with a 3.5 μg Ptx/mm^2^ coating than in those who had been treated with an uncoated standard balloon (82.2% vs. 59.5%; p<0.001) [25]. Although, these first clinical studies are showing promising results, in many patients time to repeated treatment is still too limited.

A DCB with a dose density of 6 μg Ptx/mm^2^ may be more efficacious in preventing restenosis in difficult to treat lesions by higher drug concentration in treated arterial wall.

Discussions regarding mortality after local paclitaxel application in vascular therapy [26] have not been supported by randomized trials and large real-world data [4,27–30]. It has been clearly shown that the amount of paclitaxel administered locally has no correlation with mortality [31]. In real-world data, the long-term mortality rate was even lower after DCB angioplasty of femoropopliteal lesions. Known comorbidities, risk factors, and disease severity were identified as mortality predictors but not paclitaxel [27]. Furthermore, a large meta-analysis found some evidence that improved survival was seen with coronary use of paclitaxel DCB beyond 2 years after treatment [32]. With this in mind, investigations in high-dose DCB appears to be reasonable for high-risk indications such as repeated restenosis, restenosis in DES, or AV shunts.

### Limitations

We used a model with young healthy pigs neglecting typical tissue characteristics of atherosclerotic altered arteries in humans. Nevertheless, an efficacy and safety study in peripheral arteries of pigs should be performed to evaluate safety of DCB with 6 μg Ptx/mm^2^ before starting a randomized clinical trial. Finally, only the clinical trial can provide information on efficacy and potential safety risks in patients.

## Conclusion

A novel iopromide based matrix coating for balloon catheters with a paclitaxel dose of 6 μg/mm^2^ has been investigated. The dose of 6 μg Ptx/mm^2^ was successfully as well as reproducibly coated on PTA balloon catheters. In the experimental setting, treatment with this catheter enables more than double the amount of paclitaxel transfer to internal and external iliac arteries of young healthy pigs compared to balloon catheters with 3 μg Ptx/mm^2^. Further studies concerning efficacy and safety seem to be justified.
